# Effects of sensate focus technique and position changing on sexual function of women with deep-infiltrating endometriosis after surgery: A clinical trial study

**DOI:** 10.18502/ijrm.v21i6.13638

**Published:** 2023-07-24

**Authors:** Parisa Tajik, Shadab Shahali, Roya Padmehr

**Affiliations:** ^1^Department of Reproductive Health and Midwifery, Faculty of Medical Sciences, Tarbiat Modares University, Tehran, Iran.; ^2^Reproductive Biotechnology Research Center, Avicenna Research Institute, ACECR, Tehran, Iran.

**Keywords:** Endometriosis, Sexual dysfunction, Dyspareunia.

## Abstract

**Background:**

Endometriosis is a disease that affects women throughout their sexual life. Sexual health is, therefore, a major concern for these women.

**Objective:**

This study aimed to assess the effects of the sensate focus technique and position changing on the sexual function of women with deep-infiltrating endometriosis 3-6 months after surgery.

**Materials and Methods:**

This clinical trial study was performed on 80 women with deep endometriosis, aged 18-45 yr, who were referred to the endometriosis clinic of Avicenna fertility center, Tehran, Iran for follow-up after surgery from January to September 2021. They were divided randomly into 2 groups. In the intervention group, 2-hr virtual training sessions were held and the control group only completed the questionnaires without any intervention. Sexual function was evaluated after 4 and 8 wk.

**Results:**

8 wk after the intervention, the mean score of sexual function in the intervention group was significantly improved (p < 0.001). The mean total score of sexual function in the pre-intervention period reached from 24.16 to 28.31 in 4 wk after the intervention and 29.85, 8 wk after the intervention. The mean score of sexual pain during the follow-up periods was significantly improved in the intervention group (p < 0.001).

**Conclusion:**

Sensate focus technique and sexual position changing improved sexual function in women with deep endometriosis after surgery.

## 1. Introduction

Sexual function is one of the most critical dimensions of human life (1). It can impact all aspects of a couple's lives (2). The origin of sexual dysfunction may be due to a psychological condition or a social, biological, medical, or a combination of these factors (3). Sexual dysfunction is a prevalent issue and affects 41% women of reproductive age worldwide (4). The overall prevalence of sexual dysfunction among the reproductive-age women in Iran was estimated as 52% (5). Several factors are involved in developing and emerging women's sexual dysfunction, and endometriosis is one of them (6).

Endometriosis is defined as the presence of endometrial tissue (endocrine or stroma) outside the uterine cavity (7), and there is a relatively common condition, with its prevalence reported in 15% of women of reproductive age (8, 9). Deep infiltrating endometriosis (DIE) is the most severe form of endometriosis, associated with the peritoneum or peritoneal layer with a depth of 5 mm (10).

The most common symptom of endometriosis is chronic pelvic pain, dysmenorrhea, dyspareunia, sexual dysfunction, and infertility which can significantly affect women's quality of life and mental health (11). In a study conducted on 51 Indian women with endometriosis, it was found that 47% of patients had sexual dysfunction. With increasing staging of endometriosis, the prevalence of sexual dysfunction also increased (12). The prevalence of sexual dysfunction in women with endometriosis is about 61% (13).

Dyspareunia can be observed in 60-70% of women with DIE after their surgery and can lead to sexual dysfunction, and cause negative effects in their relationships (14). Surgery can have a positive effect on endometriosis symptoms (15) but does not necessarily lead to definite solving of their sexual problems. Researchers have recently used different interventions to improve sexual activity and sexual satisfaction in women (16). The research has shown that providing couples with interventions can help them improve their relationship (17). One of the interventions is the sensate focus technique, introduced by Masters and Johnson as a couple-based intervention that includes sensual touching and cuddling (18). Some studies showed strong evidence about sensate focus technique's effectiveness in treating various types of sexual dysfunctions (19).

A study showed that some sexual positions reduce pain during intercourse. The “missionary position”, “woman on top”, the “spoon position”, and “vaginally from behind” are possible positions in intercourse despite dyspareunia in women with endometriosis (20).

Endometriosis, especially deep infiltrated lesions, disrupts the sexual function of women, which is destroyed due to pain, depression, or frustration. These problems remain even after surgery (21). Medications and surgical treatments may only suppress the symptoms, but they do not treat them. Also, due to the mechanical impact of pain in women with endometriosis and the fact that even after the surgery, the negative mental effects of the disease are present. Although surgery has a significant effect on reducing pain severity, it will be ineffective on psychological effects (22). So, we decided to investigate the effect of the sensate focus technique and sexual position changes on the sexual function of women with DIE 3-6 months after surgery.

## 2. Materials and Methods

### Study design

This parallel non-blinded clinical trial study was conducted on 80 women admitted to the Endometriosis Clinic of Avicenna Fertility Center, Tehran, Iran, 3-6 months after surgery, from January to September 2021.

### Inclusion criteria 

Married women aged between 18 and 45 yr, having sexual intercourse in the last 8 wk, not having a known underlying disease other than endometriosis, mental illness, husband monogamy and living with the spouse during the study, literate in reading and writing the Persian language, being Iranian and living in Tehran, not addicted to drugs and alcohol, had no stressful accident in a month.

### Exclusion criteria

The couple's unwillingness to stay in the study, getting pregnant while studying, failure to perform a regular intervention program, and remaining severe surgical complications (according to the research unit and the file).

### Randomization

Participants were randomly assigned to research groups using random numbers table. Paired and individual methods were used to assign samples to the intervention and control groups. Even numbers in the random number table were assigned to the intervention group, and odd numbers to the control group.

### Sampling

The sample size in terms of total sexual function score, 1 month after the intervention in the 2 control and intervention groups with a sample size of 10 people, as a pilot, was calculated for each group, using the below formula, as 36. With an estimated drop of 10%, it was calculated as 40 (80 in total). The mean of the 2 groups was 24.65 and 27.55, respectively, and the difference in standard deviation was 3.1, with power level (1-beta) = 0.8 at the alpha threshold = 0.05. 


$$n=\frac{{\left(Z_{1-\frac{\alpha }{2}}+Z_{1-\beta }\right)}^{2}\left(S_{1}^{2}+S_{2}^{2}\right)}{{\left(\mu_{1}-\mu_{2}\right)}^{2}}$$

### Data collection tools

The data collection tool in this study were female sexual function index (FSFI), which includes 19 questions that assess women's sexual function during the previous 4 wk in 6 domains of desire, arousal, lubrication, orgasm, sexual satisfaction, and sexual pain. The score varies from 2-36 (min-max). A score below 26.5 is classified as female sexual dysfunction (23). The validity and reliability of the Persian version of this questionnaire were confirmed (24). Each participant's sexual pain was assessed using visual analog scale (VAS) in addition to questions of pain domain of FSFI. The VAS scale is measured in millimeters and ranges from 0 (no pain) to 100 (worst pain). It is the most commonly used pain scale and is a valid tool for evaluating chronic pain during endometriosis (25).

### Intervention

80 women who underwent laparoscopic excision of DIE in the last 3-6 months with no hormonal medication use after surgery were included in the study. The research units explained the study's objectives and the confidentiality of the answers. After obtaining informed written consent, they completed the reproductive-demographic questionnaire, FSFI, and VAS for evaluating the primary outcomes, which is a female sexual function including sexual pain. Then they were randomly divided into 2 groups, intervention and control.

According to the coronavirus disease 2019 pandemic, a virtual training class was held for the intervention group members to learn sensate focus technique and sexual positions online through the Big Blue Button video conferencing system in a 2-hr session. The educational section content is presented in table I. At the end of the training class, the participant's questions were answered.

A step-by-step booklet on sensate focus technique (prepared by Hamdollahi et al. In 2020 and evaluated by experts and users, content validity ratio: 83%) (26) and sexual positions were provided to participants. The content of the booklet includes an introduction to men's and women's sexual health, the sensate focus technique, and instructions on how to do it, and the recommendation of some sexual positions such as spoon position, woman on top, missionary position, vaginal penetration from behind, and hot seat position (20, 27).

Participants phone numbers and addresses were recorded for access to samples and follow-up evaluations, also the researcher's phone number was made available to answer any questions the woman might have. After the end of educational sessions, the researcher contacted the research units in the intervention group every week to ensure that the sensate focus technique was performed and the best sexual position was discovered.

1 and 2 months after starting the study, the FSFI and VAS were completed by the control and intervention groups. The control group was also informed that if they wish, after completing the research, they can participate in a free workshop to teach sensate focus techniques and change positions, which none of these people have participated in to date.

**Table 1:** Descriptive statistics of quantitative demographic variables


**1.**	Introducing the anatomy and physiology of the female reproductive system
**2.**	Introducing the anatomy and physiology of the male reproductive system
**3.**	Introducing the normal sexual response cycle
**4.**	Myths and false beliefs
**5.**	Definition of endometriosis and its effect on sexual function
**6.**	Questions and answers
**7.**	ntroduction of sensate focus technique (Masters and Johnson)
**8.**	Teaching the first stage of the sensate focus technique
**9.**	Teaching the 2 ${}^{\text{nd}}$ stage of the sensate focus technique
**10.**	Teaching the third stage of the sensate focus technique
**11.**	Question and answer
**12.**	Teaching the sexual positions that can reduce sexual pain W9923

### Ethical considerations

This study was approved by the Ethics Committee in Biomedical Research of Tarbiat Modares University, Tehran, Iran (Code: IR.MODARES.REC.1399.090), documented in the Iranian registry of clinical trials, and updated on 2023-01-28. All the participants received thorough information about the study. Written informed consent was obtained from each participant.

### Statistical analysis

Data were collected and analyzed using SPSS (Statistical Package for the Social Sciences, version 26.0, SPSS Inc., Chicago, Illinois, USA). For descriptive analysis of data, absolute and relative frequency distribution indices, mean, and standard deviation have been reported. Also, for inferential data analysis independent *t* test (Mann-Whitney or Chi-square test) was used to compare the intervention and control group before and after the intervention. Analysis of variance with repeated measures (Friedman) was applied to compare before, 1 month, and 2 months after the intervention in 2 groups. P < 0.05 is considered the significant level.

## 3. Results

**Figure 1:**
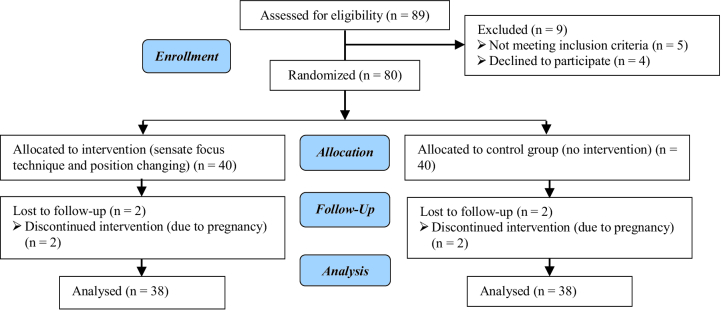
Consort 2010 flow diagram.

Out of 80 participants, 2 in the intervention group and 2 in the control group were excluded from the study due to pregnancy. Finally, the data analysis was performed on 76 women who completed the intervention (Figure 1). The mean age of women in the intervention group was 35.26 ± 5.27 yr, and in the control group was 33.71 ± 4.79 yr. The duration of marriage in the intervention group was 10.29 ± 5.37 and in the control group was 9.5 ± 5.45. The test results comparing the participants' mean age and marriage duration did not show a significant difference between the intervention and control groups (Table II).

The results showed that only the level of education of participants in the control and intervention groups showed a significant difference (Table III). However, the covariance analysis showed no significant relationship between the level of education and sexual function (before the intervention, 1 month after intervention, and 2 months after intervention) (Table IV).

According to Mann-Whitney U test results, the mean total score of sexual function in the pre-intervention period was not significantly different in the intervention and control groups. But in the period 1 month after the intervention and 2, months after the intervention, the mean total score of sexual function in the intervention group was significantly higher than the control group.

In addition, based on the results of the Friedman test, the mean total score of sexual function in follow-up periods for the intervention and control groups was significantly different (p < 0.001), but it should be noted that for the intervention group, the mean total score of sexual function in the pre-intervention period reached from 24.16-28.31 in 1 month after the intervention and 29.85, 2 months after the intervention. Still, for the control group, the mean total score of sexual function increased from 25.32 in the pre-intervention period. The intervention reached 24.49 in 1 month and 23.79 in 2 months after the intervention (Table V). The comparison of the mean scores of sexual function domains in women before, 1 month, 2 months after intervention in control and intervention groups is shown in table VI.

According to the results of the Mann-Whitney U test, the mean scores of sexual pain by VAS before the intervention and 2 months after the intervention were significantly different in the intervention and control groups. However, in the period 1 month after the intervention and 2 months after the intervention, the mean scores of pain were not significantly different in the control group. In addition, based on the results of the Friedman test, the mean score of sexual pain in follow-up periods was significantly different between the intervention and control groups (p < 0.001).

However, it should be noted that for the intervention group, the mean score of sexual pain from 3.92 in the pre-intervention period decreased to 2.66 in 1 month after the intervention and 2.26 in 2 months after the intervention. Still, for the control group, the mean score of pain was 2.63 in the pre-intervention period. It was 3.05 in 1 month after the intervention and 3.47 in 2 months after the intervention (Table VII).

**Table 2:** Descriptive statistics of quantitative demographic variables


**Variable**	**Control group**	**Intervention group**	**Total**	**P-value**
**Age (yr)**	33.71 ± 4.79	35.26 ± 5.27	34.49 ± 5.06	0.49
**Duration of marriage (yr)**	9.5 ± 5.45	10.29 ± 5.37	9.89 ± 5.39	0.66
Data presented as Mean ± SD. *t* test				

**Table 3:** Descriptive statistics of qualitative variables


**Variables**		**Control group**	**Intervention group**	**P-value**
**Education level**				
	**High school**	18 (47.37)	8 (21.05)	
	**Associate degree**	2 (5.26)	4 (10.53)	
	**Bachelor**	11 (28.95)	21 (55.26)	
	**Masters and higher**	7 (18.42)	5 (13.16)	0.04
**Spouse's education level**				
	**High school**	14 (36.84)	6 (15.79)		
	**Associate degree**	5 (13.16)	6 (15.79)		
	**Bachelor**	13 (34.21)	19 (50)	
	**Masters and higher**	6 (15.79)	7 (18.42)	0.98
**Employment status**				
	**Housewife**	28 (73.68)	26 (68.42)		
	**Employed**	5 (13.16)	10 (26.32)		
	**Self employed**	5 (13.16)	2 (5.26)	0.22
**Spouse's employment status**				
	**Worker**	5 (13.16)	4 (10.53)		
	**Self employed**	24 (63.16)	19 (50)	
	**Employee**	9 (23.68)	15 (39.47)	0.87
**Laparoscopy history**				
	**Yes**	15 (39.47)	21 (55.26)		
	**No**	23 (60.53)	17 (44.74)	0.70
**Endometriosis stage**				
	**Stage1**	3 (7.89)	7 (18.42)	
	**Stage2**	10 (26.32)	5 (13.16)	
	**Stage3**	17 (44.74)	15 (39.47)	
	**Stage4**	8 (21.05)	11 (28.95)	0.27
**Abortions (n)**				
	**0**	30 (78.95)	32 (84.21)		
	**1**	8 (21.05)	6 (15.79)		0.20
**Fertility status**					
	**Fertile**	22 (57.89)	15 (39.47)	
	**Infertile**	16 (42.11)	23 (60.53)	0.82
**Para**				
	**0**	21 (55.26)	24 (63.16)	
	**1**	17 (44.74)	14 (36.84)	0.76
**Gravida**				
	**0**	16 (42.11)	22 (57.89)	
	**1**	10 (26.32)	8 (21.05)	
	**2**	12 (31.58)	8 (21.05)	0.83
Data presented as n (%). Chi-square				

**Table 4:** Determining the direct effect of (quantitative) demographic variables on sexual function


	**Variable**	
**Women's sexual function**	**Correlation**	**Age**	**Duration of marriage**
			Pearson	-0.221	-1.29
**Before**	Significance level	0.055	0.266
		Pearson	-0.097	-0.047
**After a month**	Significance level	0.403	0.688
		Pearson	-0.047	-0.072
**After 2 months**	Significance level	0.684	0.538
	Statistical test: Pearson correlation Test (measures the statistical relationship between demographic variables on sexual function)			

**Table 5:** Comparison of the mean total score of sexual function in women before, 1 month, and 2 months after the intervention betweengroups


**Group**	**Before study**	**After a month**	**After 2 months**	P-value*
**Intervention group**	24.16 ± 5.31	28.31 ± 3.63	29.85 ± 2.93	< 0.001
**Control group**	25.32 ± 4.54	24.49 ± 4.32	23.79 ± 4.40	< 0.001
P-value**	0.53	< 0.001	< 0.001	
Data presented as Mean ± SD. *Friedman test, **Mann-Whitney U test				

**Table 6:** Comparison of the mean score of sexual function domains in women before, 1 month, 2 months after intervention between groups


**Sexual functions**		**Before study**	**Before study**	**After 2 months**	P-value*
**Sexual desire**					
	**Intervention group**	3.55 ± 1.02	4.17 ± 0.74	4.44 ± 0.68	< 0.001
	**Control group**	3.36 ± 0.95	3.24 ± 0.89	3.16 ± 091	0.019
	P-value**	0.405	< 0.001	< 0.001	
**Sexual arousal**					
	**Intervention group**	3.58 ± 0.88	4.21 ± 0.75	4.48 ± 0.64	< 0.001
	**Control group**	3.36 ± 0.95	3.24 ± 0.89	3.16 ± 0.91	0.019
	P-value**	0.74	< 0.001	< 0.001	
**Lubrication**					
	**Intervention group**	4.29 ± 1.26	4.89 ± 0.79	5.20 ± 0.60	< 0.001
	**Control group**	4.42 ± 0.86	4.26 ± 0.92	4.07 ± 0.93	0.019
	P-value**	0.58	0.002	< 0.001	
**Orgasm**			
	**Intervention group**	4.38 ± 1.43	5.02 ± 0.84	5.24 ± 0.68	< 0.001
	**Control group**	4.33 ± 1.27	4.33 ± 1.19	4.26 ± 1.20	0.121
	P-value**	0.74	< 0.001	< 0.001	
**Satisfaction**			
	**Intervention group**	4.44 ± 1.16	5.12 ± 0.86	5.39 ± 0.72	< 0.001
	**Control group**	4.78 ± 1.15	4.61 ± 1.14	4.52 ± 1.13	0.121
	P-value**	0.207	0.033	< 0.001	
**Pain**					
	**Intervention group**	4.37 ± 1.44	4.92 ± 1.06	5.09 ± 0.90	< 0.001
	**Control group**	4.91 ± 0.98	4.73 ± 1.11	4.55 ± 1.25	0.005
	P-value**	0.06	0.044	0.032	
Data presented as Mean ± SD. *Friedman test. **Mann-Whitney U test					

**Table 7:** Visual analogue scale in women before, 1, and 2 months after intervention


**Visual analogue scale**	**Before study**	**After a month**	**After 2 months**	P-value*
**Intervention group**	3.92 ± 2.84	2.66 ± 2.30	2.26 ± 2.11	< 0.001
**Control group**	2.63 ± 2.33	3.05 ± 2.63	3.47 ± 2.90	0.02
P-value**	0.03	0.48	0.04	
Data presented as Mean ± SD. *Friedman test.** Mann-Whitney U test				

## 4. Discussion

This study investigated the effect of the sensate focus technique and sexual positions on sexual function in women with deep endo 3-6 months after surgery. As a result, the mean score of total sexual function and its domains before the intervention did not differ significantly between the intervention and control groups, but 1 month and 2 months after the intervention, the mean score of sexual function in the intervention group was significantly improved compared to pre-intervention. Still, in the control group, there was a decrease in total score and all domains of sexual function, and there was a significant difference between the 2 groups. A study in Iran showed that the sensate focus technique could improve the total score of sexual function and all of its domains (26). The result of a systematic review study showed that the sensual focus technique is effective in treating women with sexual dysfunctions, such as decreased sexual desire, arousal disorders, vaginismus, dyspareunia, and anorgasmia (28) that are in line with our study results.

Also, the mean score of sexual pain in follow-up periods for the intervention group was decreased, and a higher recovery rate in the intervention group was observed. The study evaluated the quality of sexual life of women with endometriosis and reported by changing the sexual position, the pain was decreased during sexual relationships (29), which is in line with the present study.

Astudy showed that the “missionary position”, “woman on top”, the “spoon position”, and “vaginally from behind” were used during intercourse despite dyspareunia in women with endometriosis (20). This study's results were inconsistent with ours, which could be due to differences in the research participants (partners of women with and without endometriosis). A study investigated the major and minor complications after anterior rectal resection for deeply infiltrating endo and showed that there is insufficient lubrication during intercourse (36.5%) after surgery. In the present study it causes pain (30), and mental preparation learned in step-by-step exercises of sensate focus can be the basis for reducing pain. The mean score of sexual pain for the intervention group had a decreasing process, which can be due to the practice of non-sexual touch to sexual touching in sensate focus technique, resulting in loss of fear of pain, controlling the pain catastrophization in women, reducing the pain during their sexual relationship, and trained in order to use sexual positions with less pain. Still, in the control group, the mean score of sexual pain had increased, possibly due to the recurrence of lesions and surgical complications due to the deepness of endo lesions.

It should also be noted that the participants did not take the hormonal drugs after surgery, and this factor itself may imply pain. A study evaluated the deep dyspareunia 6 months after the complete removal of endometriosis using laparoscopy with the preservation of the nerve. They found a significant decrease in dyspareunia at a 6-month follow-up, but some women did not experience any improvement in their dyspareunia, and in some cases, the condition deteriorated or manifested itself for the first time (31), which can be explained by another study, which concluded that the severity of pain experienced by women who have endometriosis may be the predictor of their response to surgery (32).

The present study has not evaluated the sexual function of women's spouses, it is suggested that in future studies, the sexual function of couples be performed simultaneously, and the results be compared with each other.

## 5. Conclusion 

The results of our study show that the sensate focus technique and sexual position changing can improve sexual function in women with deep endometriosis after surgery. The practice of non-sexual touch to sexual touching in sensate focus technique with training to use sexual positions with less pain, resulting in loss of fear of pain and controlling the pain catastrophization in women, and reducing pain during their sexual relationship. Because endometriosis has a high prevalence in communities and has many negative effects on various aspects of female sexual function, chronic pelvic pain, advanced stages of the disease, and the presence of physical, mental, and psychological diseases affect the sexual function of these women. Even with current treatments, including surgery, these disorders may persist. Despite the proven effectiveness of laparoscopic surgery, the recurrence of postoperative pain symptoms remains a major challenge and an important issue in the long run. Enhancing sexual function should be regarded as the primary clinical objective of endometriosis treatment, as it not only addresses dyspareunia or pain during intercourse but also improves overall quality of life.

##  Conflict of Interest

The authors declare that they have no conflict of interest.
